# Assessing the Soundscape Appropriateness in the Vicinity of a Heliport in an Urban Park of Quito (Ecuador) Using Immersive Audio-Visual Scenarios

**DOI:** 10.3390/ijerph19106116

**Published:** 2022-05-18

**Authors:** Virginia Puyana-Romero, José Luis Cueto, Ismael Sebastián Caizapasto-Sánchez, Gabriel Eduardo Marcillo-Calispa

**Affiliations:** 1 Grupo de Investigación Entornos Acústicos, Departamento de Ingeniería en Sonido y Acústica, Universidad de Las Américas, Quito 170125, Ecuador; ismael.caizapasto@udla.edu.ec (I.S.C.-S.); gabriel.marcillo@udla.edu.ec (G.E.M.-C.); 2 Laboratorio de Fonética e Ingeniería Acústica, Instituto de Lingüistica Aplicada, Universidad de Cádiz, 11002 Cádiz, Spain; 3 Laboratorio de Ingeniería Acústica, Universidad de Cádiz, 11510 Puerto Real, Spain; joseluis.cueto@uca.es

**Keywords:** helicopter noise, soundscape, acoustic environment, quiet areas, Immersive Virtual Reality

## Abstract

Heliports are facilities that play a fundamental role in security and emergency operations. Since rotorcrafts do not need much space for take-off and landing, heliports are normally immersed in the urban fabric of our cities. However, they generate high noise levels, which can cause a nuisance, especially in outdoor areas intended for the recreation of citizens. This paper studies how helicopter noise affects the perception of the soundscape appropriateness and landscape quality in the vicinity of a heliport located in an urban park, using semantic differential scales and appraisals on the noise sources. The study area was the “Parque del Bicentenario” in Quito, Ecuador. Immersive Virtual Reality (IVR) laboratory tests using 360-degree videos and spatial audio were preferred to on-site questionnaires, given the difficulty of predicting when helicopter noise events would occur. For the statistical analysis, objective acoustic and psychoacoustic parameters have also been considered. Results show that the soundscape is perceived as more pleasant and less chaotic when there is no helicopter noise. Furthermore, with the same visual stimuli, the appraisals of the landscape are much better in the scenarios without the helicopter noise. Sharpness is the psychoacoustic parameter that best explains the variance of the subjective variables evaluated.

## 1. Introduction

Urban areas need accessible public open spaces that contribute to the improvement of the quality of life of citizens. These zones must be homogeneously distributed throughout the city, so that everybody can easily access them, and be sufficient in number and extension to satisfy the needs of the population. Urban green spaces play an important role from a social perspective by promoting physical activity, allowing rest or relaxation, increasing social interaction, and reducing social isolation in urban settings [1,2]. Regarding the acoustic quality, the European Noise Directive indicates that there must be quiet areas in cities to prevent the negative effects of noise on health; however, it does not establish quantitative criteria to define what is considered a quiet area. The European Environmental Agency (EEA) has compiled recommendations from various European countries for noise limits in quiet areas [3]. However, for urban contexts, it is difficult to establish noise limits, since L_den_ values below 45 dB are hardly ever found [3]. For this reason, the EEA recognizes L_den_ levels below 55 dB as a potential criterion for urban areas [3]. These recommendations can be applied to countries that do not have their own criteria to define what a quiet area is.

The lack of homogeneous criteria defined by the authorities to quantitatively evaluate the acoustic quality of quiet areas has led to the publication of many research studies that have chosen to qualify (and not only quantify) the acoustic environment of these recreational spaces. They have achieved this by studying the soundscape, defined by ISO 16293 as the “acoustic environment as perceived or experienced and/or understood by a person or people, in context” [4]. Soundscapes generally contain many sounds that occur simultaneously or separately in time. Despite their complexity, soundscapes can be evaluated through the perception of the set of momentary or past sensations that an individual has [5]. According to the ISO 16293 definition, the study of soundscapes needs citizen participation for its evaluation. It, therefore, seems that conducting in situ surveys is a good instrument for evaluating these landscapes [6]. However, the evaluation of certain sound events is difficult in on-site surveys, as they may or may not occur during the survey development. In such cases, the use of laboratory tests seems more appropriate, which allows for reproducing the same acoustic and visual conditions for all participants, and therefore, more control of the study settings.

One of the most suitable methods for this type of test is based on the use of Immersive Virtual Reality (IVR) environments since they allow participants to be surrounded by scenarios that simulate the real world. The reproduction of 360-degree videos and spatial audio using IVR tools in acoustic research enables a balance between realism and ease of test setup, which makes them ideal for diagnosing the problems of existing acoustic environments. In a recent study by Puyana et al., the ecological validity of 360-degree videos and spatial audio was evaluated using an IVR headset [7]. Semantic differential analysis of the acoustic environment and a question about the soundscape quality were analyzed, showing no significant differences between the responses given to the on-site survey and the IVR tests. This study serves as an endorsement for carrying out this type of test [8,9,10]. Similar analyses of the ecological validity of virtual reality in the evaluation of soundscapes were also carried out for scenarios completely simulated by computers, obtaining also very good results for the semantic differential analysis of the soundscape [11,12,13]. 

Among the different types of unwanted/intrusive sounds in parks located in urban areas, traffic noise is the most remarkable one [14,15], but there are others related to human activities, such as noise coming from construction works, unmanned aerial vehicles [16,17,18], or overflying aircraft [19,20,21], that are also very disturbing. Regarding aircraft, not many articles were found on how the noise that they produced is perceived in outdoor urban areas. Memoli et al. evaluated the perception of the dimensions of an airplane according to the noise that it emits, leading to the conclusion that, for the same flying height, louder planes are perceived as lower, but not as larger [22]. Lugten et al. evaluated the effectiveness of water sound and vegetation on masking fixed-wing aircraft noise [20], leading to the conclusion that these elements improve the perception of the pleasantness and eventfulness of the acoustic environment, although their effects differ between locations and for different sound pressure levels. Gerolymatou et al. applied a methodology in two airports in Greece based on the evaluation of the WHO’s Disability Adjusted Life Year (DALY) metrics and soundscape characteristics for the improvement of the acoustic quality of areas affected by aircraft noise [19]; according to the results, they concluded that one of the airports had to be relocated and recommended that a noise action plan be undertaken. 

The noise generated by helicopters is different from that generated by fixed-wing propeller-driven aircrafts. For propeller-driven aircrafts, the propeller axes are parallel to the direction of motion of the aircraft [23]. In the helicopter, however, the axes of rotation are normal to the direction of the flight. The rotor noise is the sound source that contributes the most to the helicopter noise, although there are some other noise sources, such as the engine, the compressor, combustion, turbine, or aerodynamic noise [23,24]. 

In helicopters, the rotor provides lift without the need for horizontal displacement, which favors take-offs and landings in relatively small spaces compared to those required by fixed-wing propeller-driven aircraft. In addition, its great maneuverability and ability to remain static in the air make it especially useful in security operations, health emergencies, or rescue. This versatility means that there are heliports immersed in or very close to the urban fabric, although they are problematic facilities from an environmental point of view. Even though the number of complaints received due to airport noise is greater than that of heliports, the noise generated in the latter type of facilities is considerable and is beginning to generate concern in the competent authorities, mainly due to the nuisance in nearby homes produced by the noise of overflight, landing, and take-off operations [25,26]. In fact, there are laws that specifically limit the noise levels of heliports for different land use categories [27], and include specific methodologies for the calculation of the helicopter noise [28]. Furthermore, several studies report that, at a given A-weighted SPL, helicopter noise is considered more annoying than that of fixed-wing aircraft [29,30]. Other studies suggest that the reaction to helicopter noise is not only related to the absolute level or type of noise but also to other factors, such as the distinctive characteristic of the acoustic signature or visual stimuli [24]. Fastl et al. [31] evaluated, through simulations, the noise emitted by a helicopter at six equidistant positions from the tail rotor using psychoacoustic parameters. Results evidenced that the directivity pattern of psychoacoustic parameters varies around the helicopter.

Although helicopter take-off and landing operations are very noisy and may diminish the rest and recreation of citizens, there is a lack of research on how they are perceived by the users of open urban areas. This study is intended to contribute to the knowledge of the acoustic perception in the vicinity of urban park heliports, using IVR combined with 360-degree videos and spatial audio.

The objective of the present study was to evaluate whether there are significant differences between the visual and acoustic perception in the vicinities of a heliport—located in an urban park—with and without helicopter noise. The “Parque del Bicentenario” of Quito was chosen as the study area. A comparison between three locations was conducted, to evaluate the influence of the landscape, distance, and type of noise on the perception of the appropriateness of the soundscape, with the helicopter on and off.

The following hypotheses were formulated to develop the study:

**H1.**  *The pleasantness of the sound sources is perceived the same with (WH) and without the helicopter noise (WOH) (sound sources).*

**H2.**  *The pleasantness of the different sound sources is perceived the same at the three locations under study (sound sources and locations).*

**H3.**  *Semantic differential scale (SDS) ratings on the soundscape are the same WH and WOH (SDS).*

**H4.**  *Semantic differential scale (SDS) ratings on the soundscape are the same at the three locations under study (SDF and locations).*

**H5.**  *General appraisals about the landscape and soundscape quality, and the frequency of visits, are the same WH and WOH (soundscape/landscape/frequency of visits).*

**H6.**  *General appraisals about the landscape and soundscape quality, and the frequency of visits, are the same at the three locations under study (soundscape appropriateness/landscape/frequency of visits and locations).*

**H7.**  *There is no association between objective and subjective acoustic parameters collected in the study (objective acoustic parameters).*

## 2. Materials and Methods

Due to urban growth, the old Mariscal Sucre International Airport of Quito was moved to the outskirts of the city, leaving the land and buildings that it occupied vacant. This land became an urban park named “Parque del Bicentenario”. The resources and the strategic location of the area meant that the airport facilities were reused, and public institutions such as the National Police and the Fire Department were established there. Both institutions have heliports to ensure the safety and wellness of citizens. The park is surrounded by “Luis G Tufiño” Avenue to the north, “Sancho Hacho”, “Rafael Aulestia”, and “Real Audiencia” avenues to the east, “Isaac Albeniz” Avenue to the south, and the backs of different buildings to the west. It has 324.72 hectares, with land used for social and public service facilities. 

According to the noise map of Quito, developed at the Universidad de Las Américas [32], the Bicentennial Park has traffic noise levels below 50 dB on a large part of its surface, and would therefore be an area to be protected—according to international recommendations—due to its extension and restorative potential for citizens. However, the operations conducted in the existing heliports may alter these possible benefits.

Three locations (L1, L2, and L3) were considered at the “Parque del Bicentenario” for the registration of the 360-degree videos. They were outside the fenced enclosure of the National Police Station Heliport. The selection of the locations was made to have a combination of different sound pressure levels and types of noise. 

The National Police of Quito has an Airbus H125 (AS350 B3e) helicopter. The Airbus H125 is a single-engine helicopter with a capacity of 6 people and a take-off power of 847 SHP.

**Figure 1:**
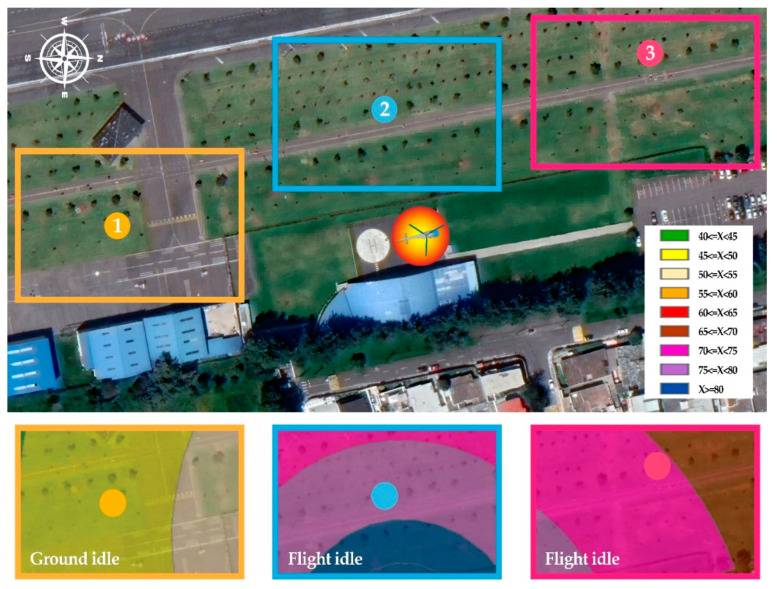
Study areas (L1, L2, and L3), sound source location, and predicted noise levels at two operational conditions.

To describe the acoustic impact generated by the helicopter and select the locations, a series of noise maps have been generated. For its development, the database contained in the Aviation Environmental Design Tool (AEDT) was used. The model selected was the Airbus AS350 Ecureuil. The acoustic model was built with the software CadnaA, considering the helicopter as a fixed sound source, since most of the measurements would be conducted with the helicopter turned on but before taking off. Two different operational phases were used for the calculations, ground idle and flight idle. Flight idle is when the helicopter is on the ground but operating at a high power setting that is approximately the same power setting used for hover operations [33,34,35]; ground idle is a previous phase with lower power settings. The predicted noise levels at both operational conditions and the locations selected are shown in Figure 1. 

Different acoustic environment settings could be recorded since the heliport managers expressly authorized the helicopter to be turned on for approximately 15 min to perform the measurements. This allowed the engine noise to be recorded during the ground idle phase (L1), and after (L2 and L3), when the helicopter was in a fixed position. At the end of the recording at L3, the helicopter took off and flew away. Consequently, most recordings were made when the helicopter was on, in the pre-take-off phase—flight idle; this is the operating condition when the noise is most perceived inside the park. 

The distances of L1, L2, and L3 to the noise source were 141.51 m, 60.36 m, and 139.60 m, respectively. It should be noted that, although the distances were similar, the type of noise at L1 (ground idle) and L3 (flight idle) was different. The audio-visual scenarios were first recorded with the sound source turned on and subsequently with the sound source turned off.

The Ricoh Theta V camera and the Ricoh TA-1 microphone were used for recording the 360-degree video images and the spatial audio, respectively. They were located at 1.65 m height, according to the recommendations of the ISO 12913-2 [36]. The Ricoh Thera V allows the recording of video in 4 K format (3840 × 1920, 56 Mbps), and the Ricoh TA-1 is an external microphone developed by Audio-Technica, equipped with 4 capsules to record sounds from 4 different directions, capturing acoustic spatial information at low and high frequencies. These devices are an economical solution compared to other devices with high technical specificity [7] but not easily accessible for local or regional administrations that may carry out this type of study. The duration of the recordings at each location was 120 s. 

During the audio and video recordings, sound pressure levels were simultaneously measured using the class I sound level meter CESVA SC310. Calibrations were carried out before and after the measurements, using the CESVA CB006 calibrator.

The “mp4” files recorded with the Ricoh Theta V camera and the TA-I microphone were transformed into a “mov” file with 4 signals in a single audio channel using the Ricoh Theta Movie Covert application. 

The original audios recorded at each location were equalized—according to the sound level meter data—so that they had a frequency content similar to that recorded on-site, using the Neumann KU100 Dummy Head, the Sennheiser HD 380 Pro headphones, and the software Protools. The reproduction chain to be used during the IVR test was set so that the A-weighted sound levels measured with the dummy head were similar to those obtained in the field measurements, with a tolerance of ±2.0 dBA. 

The images of the sound level meter and the tripods that held the devices were deleted using video editing. Audio and visual scenarios for the IVR were embedded using the Vizard 5.2 software. The IVR tests were conducted in a room with average background noise below 35 dB, using the Oculus Rift S PC-Powered VR Headset and the Sennheiser HD 380 Pro headphones. 

Acoustic measurements were post-processed to calculate psychoacoustic parameters (loudness, sharpness, fluctuation strengths, and roughness), and equivalent sound level pressure levels (linear, A- and C-weighted) were obtained from the registered files. All the psychoacoustic metrics were calculated using the software DBFA Suite.

### 2.1. Oculus Rift S Survey

**Figure 2:**
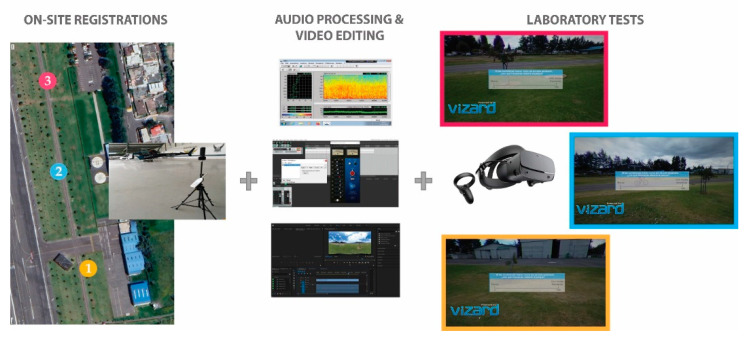
Workflow for the development of the laboratory tests.

The ISO 12913-2 Standard was taken as a reference to design and prepare the questionnaire used during the IVR tests. This standard recommends some criteria for the data collection concerning studies, research, and applications of soundscapes. The test developed had two distinguishable parts, and each part had a different questionnaire to answer; during the first part (preliminary test), participants read the instructions of the test, and filled in the informed consent form and a short socio-demographic questionnaire. During the second part (IVR test), participants were asked to put on the head-mounted display to start exploring the IVR scenarios. An example of each type of question and the different possible answers were shown to participants at the beginning of the second questionnaire. During the IVR test, participants were also asked to read and answer the questions aloud so that they were aware of the noise levels of the scenarios. The questions were shown on the head-mounted display for audio-visual scenario evaluation (Figure 2).

The first questionnaire was designed to acquire the relevant sample features and contained questions about gender, age, and hearing impairments. 

The second questionnaire was conceived to obtain information on how the acoustic and visual environment of the IVR scenarios were perceived. It included questions to describe the acoustic environment—using semantic descriptors—and about the pleasantness of sound sources. In previous studies, this research group has used semantic differential analysis with paired “opposite” adjectives. However, bipolar adjective pairs raised the question of the exactitude of the antonym labels (e.g., is chaotic the opposite of calm?) [37]. Thus, one of the problems of using bipolar opposite adjectives is that, sometimes, it is not possible to find perfectly matched pairs. For example, Chouard and Hempel [38], in a study about perfect antonyms that evaluated 242 adjectives, found paired matches in only 23% of cases. To solve this problem, unipolar scales were used to assess the suitability of the factors to describe the soundscape—in particular, the scales of the two-dimensional model developed by Axelsson et al. [5] (pleasant, chaotic, exciting, uneventful, unpleasant, calm, eventful, monotonous). Seven categories were used to evaluate the pleasantness of the sound sources, namely traffic (e.g., light and heavy vehicles), aircraft (e.g., overflying planes, helicopters), engine (e.g., construction machinery and electric generators), human (e.g., laughter and conversations), natural (e.g., wind and rain), animal (e.g., barks and birds singing), and other sounds. Examples of sounds for each category were written in the questionnaire. The questionnaire evaluated also the visual environment—as it can significantly affect the perception of an acoustic environment—through a question on the quality of the landscape. Finally, participants were asked about the frequency in which they would visit the park if the conditions were similar to those in the virtual reality scenario experienced. The rated responses ranged from 0 to 100 for unipolar scales (following the same scale range used by Axelsson et al. for the two-dimensional model for the soundscape perception [5]), and from –100 to 100 for bipolar scales.

For each location, two scenarios were evaluated: one in which the helicopter noise was predominant (WH), and the other with no predominant sound source (WOH). As there were three locations (L1, L2, and L3), six audio-visual scenarios were evaluated by each participant. The duration of each scenario was approximately 90 s. If a participant needed more time to respond, the audio-visual scenario was repeated in a loop. It should be noted that at location L3 WH, the helicopter was on and in a fixed position for 30 s, and then took off and moved away until it was no longer visible. IVR scenarios were randomly shown to participants, following a balanced Latin square design, in order to prevent sequential contraction biases in the responses [39]. The questions and the scales used in the second part of the survey can be seen in Appendix A.

In total, 26 participants took part in the study, exceeding the minimum of 20 suggested by the ISO 12913-2 Standard for the on-site evaluation of soundscapes. An equal number of men and women participated in the study. The distribution of the participants split by age and gender is shown in Table 1. None of the participants reported hearing problems. 

The equivalent A-weighted sound pressure level for the scenarios L1, L2, and L3 was 52.5 dB(A), 80.5 dB(A), and 61 dB(A), respectively, WH, and 46.6 dB(A), 39.1 dB(A), and 43.1 dB(A) WOH. Higher noise levels WOH at locations L1 and L3 were probably due to the presence of children playing basketball and a secondary street near these locations, respectively.

### 2.2. Statistical Analysis Description

SPSS statistics software and R were used for data analysis. The Shapiro–Wilk normality test was calculated to determine the distribution of the variables. Levene’s homogeneity test was used to evaluate the equality of variances for a variable calculated for repeated measures. These two tests were applied to the semantic differential scales (SDS) and the appraisal scales on the sound sources to evaluate the feasibility of applying a non-parametric test for analyzing the data. 

The distribution of the data was analyzed using violin plots. Violin plots show the distribution of the data, through a combination of a boxplot and a probability density function. The width of the curvilinear shape describes how frequently the values occur in the data set. 

Correlation analysis was conducted to evaluate the association between participants’ appraisals of the soundscape and acoustic and psychoacoustic parameters. Cohen’s convention was used to interpret the effect size of the correlations [40]. Following this criterion, a correlation coefficient between 0.10 and 0.29 represents a small association, between 0.30 and 0.49 a medium association, and 0.50 and above a large association or relationship. Further descriptive analyses were conducted on the pleasantness of the sound sources and the SDS; their results are described in Appendix B.

The Wilcoxon signed-rank test was used to determine if the repeated measures conducted (in different audiovisual conditions) were considered differently by participants. It is normally used when the dependent variable is ordinal but can be also used with continuous data that have violated the assumptions to run one-way ANOVA with repeated measures. The effect size can be used to determine the magnitude of differences between two repeated measures, and it is very useful when interpreting the results [41]; a large effect size means that the difference between variables is important [42]. Rosenthal’s expression was used to calculate the effect size [43]. Cohen’s convention was also used to interpret the effect size of the Wilcoxon signed-rank test.

According to the results of the study, and to delve into the behavior of each of the variables, Spearman’s correlations were calculated between all possible pairs of SDS, resulting in an 8 × 8 matrix (with unity in the diagonal). This matrix was subjected to principal component analysis. Similarly, a 7 × 8 matrix was built with the Spearman’s correlation coefficients calculated for each pair of noise source category and SDS and a principal component analysis was also conducted. 

## 3. Data Analysis and Results

The Shapiro–Wilk normality test was applied to assess whether the distribution of the variables was normal. In most cases, the *p*-value was less than 0.05, which indicates that there are very few variables with normal distribution. The homogeneity of variances was also studied for all repeated measures of the same factor in scenarios WH and WOH (for the evaluation of noise sources and the SDS), and also of the same factor in different locations, showing that there was no homogeneity of variances for all pairs of variables evaluated. Given the results obtained in the Shapiro–Wilk and Levene tests—as two of the assumptions for calculating repeated measures ANOVA were violated—it was decided to use non-parametric analysis to evaluate the existence of statistically significant differences between the variables. However, before analyzing the differences between groups of responses, descriptive statistics were used to summarize the characteristics of the data set.

Table 1 shows the responses of the participants who gave negative, neutral, and positive appraisals of the appropriateness of the soundscape, split by age and gender. Considering that the desirable appraisals of the soundscape of a park would be positive, the responses have been divided into two main groups that consider not-positive (which comprise negative and neutral ratings) and positive appraisals. The responses were split according to five age groups (18–24, 25–34, 35–44, 45–54, and 55–66). Furthermore, the table considers 78 responses for each condition (WH and WOH)—although there were 26 participants—since the results are shown for the three locations of the study (L1, L2, and L3).

Overall, 82.06% of the men’s appraisals about the appropriateness of the soundscape for the scenarios WH were negative or neutral, in comparison with 51.28% for those of the women. For the scenarios WOH, the percentage of men’s and women’s negative appraisals was very similar (12.82% and 10.24%, respectively). Regarding the age and the scenarios WH, the group from 18 to 24 years old seemed to give more negative or neutral appraisals than the others (74.00% in comparison with 66.68%, 50%, 50%, and 66.68% for the groups 25–34, 35–44, 45–54, and 55–66, respectively).

**Figure 3:**
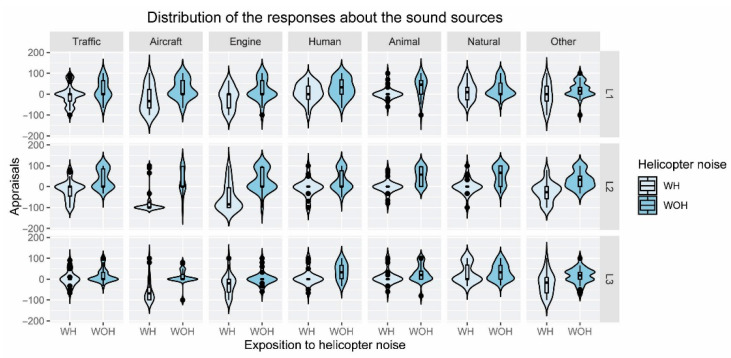
Violin plot of the responses given to the pleasantness of the sound source categories for the three locations under study (L1, L2, and L3), split into two acoustic conditions, with (WH) and without helicopter noise (WOH).

Figure 3 shows the distribution of the appraisals of the pleasantness of the different sound sources WH and WOH using violin plots. For locations L1 and L2, the medians of the traffic noise evaluations WH and WOH are similar; however, the first and third quartiles are completely different. For scenarios WOH, the first quartile is very close to the median, while, for scenarios WH, it is the third quartile that is close to the median. The same happens for the categories of engine noise and natural sound at location L1. For the other noise sources the medians are also similar or slightly lower WH.

For the scenarios WOH and the categories of traffic, aircraft, and other sounds—which are types of noise that, a priori, could be considered negative—most of the scores are close to 0 (a score that coincides with the median). Likewise, for this type of scenario, a greater concentration of responses can be appreciated around the median for human and natural sounds, being higher than for the rest of the sources. In scenarios WH, a higher number of ratings occurred for 0; however, for the aircraft noise, this happens for negative values.

**Figure 4:**
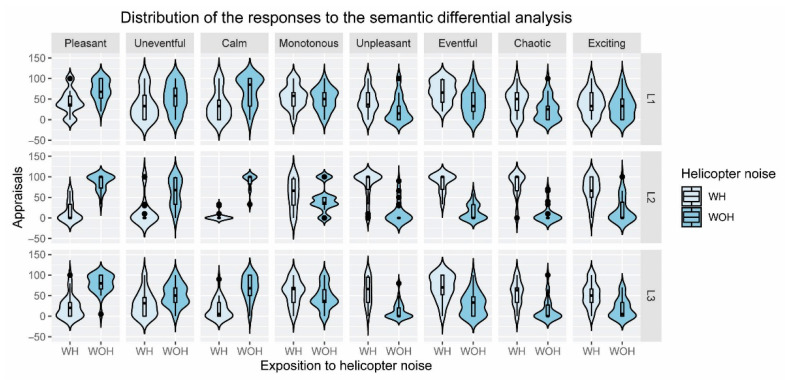
Distribution of the responses to the semantic differential analysis (pleasant, uneventful, calm, monotonous, unpleasant, eventful, chaotic, and exciting) with (WH) and without (WOH) the helicopter noise, split by location (L1, L2, and L3).

The opposite distribution of the appraisals given to the SDS-Pleasant WH and WOH can be observed in Figure 4 for locations L2 and L3. For example, for the WH scenario in location L2, the median is close to 0, and the highest concentration of responses also occurs for this value, with the median and the first quartile being very close. However, for the WOH scenario, the median is close to 100, with a higher concentration of responses close to this value and the median coinciding with the third quartile. Similarly, for other scales with positive connotations or related to tranquility (SDS-Uneventful and SDS-Calm), the median of the WH scenario is lower than the median of the WOH scenario for the three locations considered. For scales that can be considered unpleasant or disturbing (SDS-Unpleasant, SDS-Eventful, SDS-Chaotic), the trend is completely the opposite. For the SDS-Monotonous and SDS-Exciting, no clear trend can be identified for the distribution of the responses, nor concerning the locations or the WH and WOH conditions.

Wilcoxon signed-rank and Spearman’s correlation tests were used to verify the hypotheses of the study. The calculations undertaken, ordered from H1 to H7, are described below, together with the results.

### 3.1. Hypothesis H1

Hypothesis H1 was formulated to assess whether there are differences in the perceived pleasantness of the sound sources for the scenarios WH and WOH. The Wilcoxon signed-rank test can be used to compare two repeated measurements on a single sample, assessing whether their population means’ ranks differ and whether the ranked values of one group are consistently higher or lower than the other. Actually, the test was used to compare whether the perception of the sound sources in two different acoustic environments—WH and WOH—differs (Table 2). It was conducted on all the possible paired combinations of audio-visual scenarios for each perceived sound source category (seven), at locations L1, L2, and L3. The effect size was calculated applying the Rosenthal formula [43], by dividing the absolute standardized z score by the square root of the number of subjects (last column of Table 2).

As reported in Table 2, the differences between the responses of the scenarios WH and WOH are statistically significant at a 95% confidence level at least at one location for all the sound sources. In particular, there are statistically significant differences at all the locations for the categories of aircraft (*p*-value L1 = 0.049, *p*-value L2 = 0.000, *p*-value L3 = 0.000), engines (*p*-value L1 = 0.031, *p*-value L2 = 0.000, *p*-value L3 = 0.008), humans (*p*-value L1 = 0.023, *p*-value L2 = 0.011, *p*-value L3 = 0.022), and animals (*p*-value L1 = 0.049, *p*-value L2 = 0.000, *p*-value L3 = 0.036). It can be observed that, at location L2, there are statistically significant differences for all paired combinations within the same sound source category. If medians are compared for the category of animal sounds, 94.5% of participants—who gave different ratings to each compared scenario—consider that the sound of animals is more pleasant when the helicopter noise is not perceived. Similarly, the natural and human sounds are perceived as more pleasant for 67.98% and 66.63% of participants, respectively, WOH. The same trend was observed for all the sound sources analyzed, even if they are associated with artificial sounds (road traffic, aircraft, and engine noise). According to Cohen’s classification, the effect size is considered small (>0.3) only for the sound categories of “traffic” at L3 and “natural” at L3. For the other paired variables, it is considered moderate (0.3–0.5) or large (>0.5).

### 3.2. Hypothesis H2

**Figure 5:**
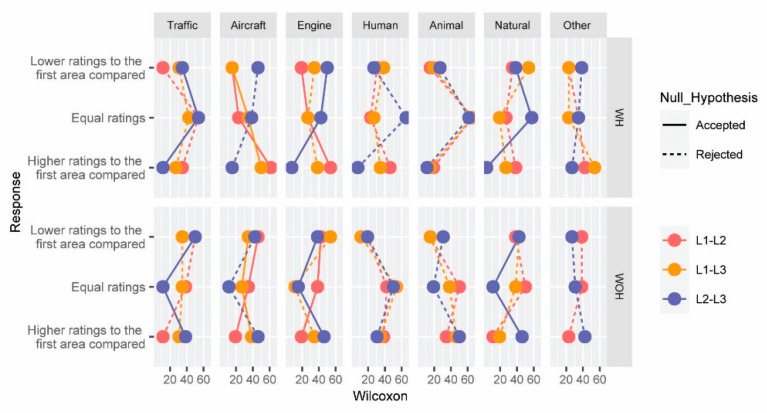
Wilcoxon signed-rank test results of the appraisals about the sound sources (sounds of road traffic, aircraft, engine, human, animal, natural, and others) at paired locations L1–L2 (red), L1–L3 (green), and L2–L3 (blue), for the conditions with and without the helicopter noise, independently considered. Significant (continuous line) and non-significant differences (dashed line) between paired variables are also represented.

This hypothesis deals with evaluating whether there are statistically significant differences in the appraisals of the sound sources at paired locations L1–L2, L1–L3, and L2–L3, for the conditions WH and WOH independently considered. Again, the Wilcoxon signed-rank test was used to calculate significant differences between the different locations. Figure 5 shows the results for each sound source. The vertical axis was divided into lower, equal, and higher ratings for the first scenario evaluated. The horizontal axis shows the percentage of participants, and it is split according to the sound source categories. 

The median differences are not statistically significant for the categories of humans, animals, and other sounds at any of the paired locations compared for both conditions, WH and WOH. For the noise category of road traffic, there are statistically significant differences only between the paired locations L2 and L3 (*p*-value = 0.023). In this case, for the scenario WH, 76.92% of the participants (excluding equal ratings) gave a higher value to location L3. WOH, although there are statistically significant differences between both locations in the perception of traffic noise (*p*-value = 0.023), only 52.94% of participants gave higher ratings to location L3—and, therefore, an opposite trend to WH can be observed. 

The evaluation of the category of aircraft noise allows for determining in which location the helicopter noise is perceived as more pleasant. For example, for L1 and L3, despite being at very similar distances from the helicopter location, a percentage of 76.47% give higher ratings to L1 (excluding equal ratings), since the level of noise in the ground idle phase is lower. Comparing L1 and L2, also a higher number of participants think that the noise of the aircraft is more pleasant in L1 (ground idle phase) than in L2 (location closest to the sound source, during the flight idle phase). 

The effect size is small for all the paired variables evaluated in this hypothesis.

### 3.3. Hypothesis H3

Hypothesis H3 was formulated to assess whether there are differences in the appraisals given to each SDS for the scenarios WH and WOH. Again, the Wilcoxon signed-rank test was conducted on all the possible paired combinations of audio-visual scenarios for each perceived SDS, at locations L1, L2, and L3.

As reported in Table 3, 21 of the 24 paired combinations show statistically significant differences at a 95% confidence level, except for SDS-Uneventful at L1 (*p*-value = 0.069) and SDS-Monotonous at L2 (*p*-value = 0.218) and L3 (*p*-value = 0.348).

For the SDS-Pleasant, the differences in the appraisals given to the three scenarios are very similar. At location L1, 95.29% of the participants (excluding equal appraisals) consider that the acoustic environment is more pleasant WOH (*p*-value = 0.000). A similar trend can be found for positions L2 and L3, with 100% (*p*-value = 0.000) and 95.88% (*p*-value = 0.000), respectively.

At L1, the number of people who gave lower ratings for the SDS-Pleasant and the scenario WH is slightly smaller than at L2 and L3. At this location, participants gave also a lower rating for the SDS-Uneventful and SDS-Calm to the scenario WH (88.72%) than at locations L2 (100%) and L3 (90.47%) (excluding equal ratings). Similarly, more participants gave equal ratings at L1 to the SDS-Pleasant (19.2%), SDS-Uneventful (26.9%), and SDS-Calm (30.8%) than to locations L2 and L3. 

Most participants gave high ratings to the scenario WH for SDS-Monotonous, SDS-Unpleasant, SDS-Eventful, SDS-Chaotic, and SDS-Exciting. For example, for SDS-Unpleasant, 76.9%, 92.3%, and 69.2% gave higher ratings (to the scenario WH) at locations L1, L2, and L3, respectively. 

The effect size is large for SDS-Pleasant, SDS-Uneventful, SDS-Calm, SDS-Unpleasant, SDS-Eventful, and SDS-Chaotic at the three locations studied. For the other SDS, the effect size is moderate or large, except for the SDS-Monotonous at L3, which is small.

### 3.4. Hypothesis H4

**Figure 6:**
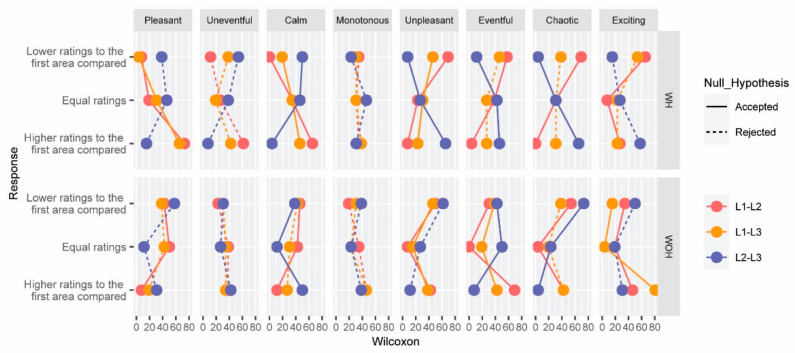
Wilcoxon signed-rank test results for each of the possible combinations of locations—L1–L2 (red), L1–L3 (green), and L2–L3 (blue)—for the eight SDS under study (pleasant, uneventful, calm, monotonous, unpleasant, uneventful, chaotic, and exciting). Results are split by the scenarios with (WH) and without the helicopter noise (WOH). Significant (continuous line) and non-significant differences (dashed line) between paired variables are also represented.

This hypothesis deals with evaluating whether there are statistically significant differences in the SDS of the acoustic environments at paired locations L1–L2, L1–L3, and L2–L3, for the conditions WH and WOH independently considered. Figure 6 shows the results of the Wilcoxon signed-rank test for the mentioned paired variables. The vertical axis was split into lower, equal, and higher ratings for the first scenario evaluated. The horizontal axis, however, was divided into the SDS, and it shows the percentage of participants. 

For the SDS-Uneventful and SDS-Monotonous, there was no pair of locations compared with statistically significant differences for both conditions considered (WH and WOH). For the SDS-Calm and SDS-Unpleasant WH, all the paired locations considered are statistically significant. For example, more participants give lower ratings at the SDS-Unpleasant at L1 (where the helicopter was in the ground idle phase) than at the other locations (7.69% for the comparison with L2—*p*-value = 0.00, and 23.08% for the comparison with L3—*p*-value = 0.044). From the comparison between the acoustic environments at L2 and L3, it can be inferred that L2 is considered more unpleasant than L3. The same trend can be observed for the scales of SDS-Eventful, SDS-Chaotic, and SDS-Exciting, presenting statistically significant differences in all the paired locations compared. The opposite trend can be observed for the SDS-Pleasant and SDS-Calm, with higher ratings at location L1 than at L2 and L3.

For the SDS-Pleasant and SDS-Calm and the scenarios who (as opposed to the scenarios WH), most of the participants give lower ratings to the L1 area than to L2 and L3.

The effect size for the SDS-Exciting, SDS-Calm, and SDS-Unpleasant is moderate or large at all the paired locations. The effect size of the other variables is small for one of the three paired locations (E _Pleasant L2–L3_ = 0.30, E _Chaotic L1–L3_ = 0.28, E _Uneventful L1–L3_ = 0.12, E _Eventful L1–L3_ = 0.12), but not for the SDS-Monotonous (the three paired combinations present a small effect size).

### 3.5. Hypothesis H5

Hypothesis H5 was formulated to evaluate whether there are differences in the appraisals given to each general question (soundscape appropriateness, landscape quality, and the frequency of visits) for the scenarios WH and WOH. The results of the Wilcoxon signed-rank test for this hypothesis are shown in Table 4.

There are significant differences for all the paired variables evaluated to test Hypothesis H5. Participants consider the soundscape more appropriate in the places in the scenarios who (17, 23, and 21 participants at locations L1, L2, and L3, respectively). Similar observations are made for the landscape quality and the frequency of visits. The effect size is large for all the paired variables but the landscape quality and the frequency of visits at L2.

### 3.6. Hypothesis H6

**Figure 7:**
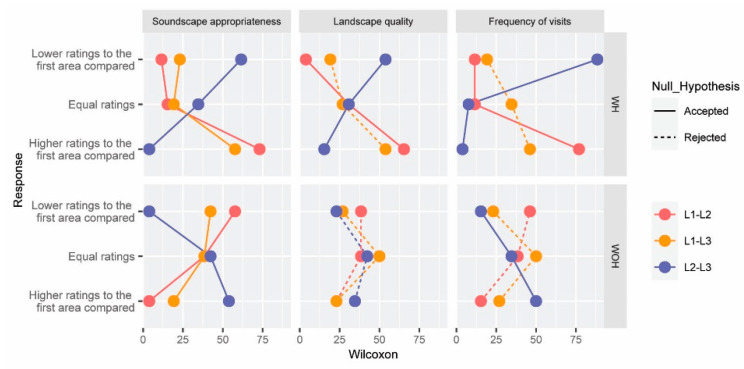
Wilcoxon signed-rank test results for each of the possible combinations of locations—L1–L2 (red), L1–L3 (green), L2–L3 (blue)—on the appraisals given to the soundscape appropriateness, visual quality, and frequency of visits. Results are split by the scenarios with (WH) and without the helicopter noise (WOH). Significant (continuous line) and non-significant differences (dashed line) between paired variables are also represented.

Hypothesis H6 was formulated to validate whether there are statistically significant differences in the perception of the soundscape appropriateness, landscape quality, and frequency of visits between the three possible paired locations (L1–L2, L1–L3, and L2–L3). The results of the Wilcoxon signed-rank test for the mentioned paired variables are shown in Figure 7. The vertical axis was split into lower, equal, and higher ratings for the first scenario evaluated. The horizontal axis, however, shows the percentage of participants, and it was divided into the soundscape appropriateness, landscape quality, and frequency of visits.

For WH, although the three locations are similar according to the landscape features, the soundscape of L1 is considered more appropriate than that of L2 (with a significance of 0.05) when the sound of the helicopter can be heard. Similar observations are made with the frequency of visits (people would visit more frequently L1 than L2). However, without the helicopter noise, the landscape quality and the frequency of visits are higher at L2 than at L1.

When comparing the statistical tests conducted for L2–L3, the trends are opposite for the scenarios WH and WOH (even for the landscape quality). The results for L1–L3—comparing the presence or absence of the helicopter noise—are also opposite for the appropriateness of the soundscape and the landscape quality. For the frequency of visits, however, WOH, the number of participants who gave lower ratings to L1 (than to L3) is similar to those who gave it higher ratings.

### 3.7. Hypothesis H7

Hypothesis H7 was formulated to verify if a relationship between the objective acoustic parameters and the subjected acoustic variables evaluated can be established.

Table 5 shows Spearman’s correlation between the acoustic and psychoacoustic parameters (LZ_eq_, LA_eq_, LC_eq_, Loudness, Sharpness, Roughness, and Fluctuation Strength), and the subjective ratings on the soundscape (sound sources, SDS, soundscape appropriateness, landscape quality, and frequency of visits). According to Cohen’s criterion, there is a small association—although significant at the 0.01 level—between aircraft noise and loudness (r = −0.22) and a moderate association with sharpness (r = −0.32). Aircraft noise is the sound source with the strongest negative correlation (the lower the equivalent noise level of the helicopter, the higher its pleasantness) with LZeq (r = −0.58), LCeq (r = −0.59), and LAeq (r = −0.59). 

Regarding the SDS, the highest correlation coefficients occur between the SDS-Calm and the acoustic parameters (r_LZeq_ = −0.70, r_LCeq_ = −0.74, r_LAeq_ = −0.74).

Sharpness is the psychoacoustic parameter that shows a higher correlation with all the sound sources—traffic (r = −0.19), aircraft (r = −0.32), engine (r = −0.23), animal (r = −0.22), natural (r = 0.2), and other (r = 0.25)—although the correlation is not significant with the human category. A similar trend can be found for all the SDS, but not with SDS- Monotonous, with a moderate positive association with SDS-Chaotic (r = 0.41), SDS-Unpleasant (r = 0.42), and SDS-Eventful (r = 0.45), and a moderate negative association with SDS-Pleasant (r = 0.49) and SDS-Calm (f = 0.45). When evaluating the general questions of the acoustic environment and the sharpness, a strong negative association was found with the appropriateness of the soundscape (r = −0.40) and the willingness to return to the park (r = −0.39), and a moderate negative association with the landscape quality (r = −0.24). 

The strongest associations of fluctuation strength and roughness occur with the SDS-Eventful, with a correlation coefficient of −0.25 and 0.24, respectively.

## 4. Discussion

This study evaluated whether different acoustic and visual aspects are perceived the same with and without the presence of helicopter noise, and in different locations, near a heliport at the Parque del Bicentenario, in Quito. In order to achieve this, the Wilcoxon signed-rank test was conducted for the pleasantness of the sound sources and for different SDS. The correlations between acoustic and psychoacoustic parameters with the subjective ratings on the soundscape appropriateness and landscape quality were also evaluated. Different hypotheses were assessed in order to identify how the soundscape and landscape are perceived in the vicinity of a heliport. 

For the scenarios WOH and the categories of traffic, aircraft, and other sounds, most of the scores were close to 0 (a score that coincides with the median). This may be because they were sounds that were barely perceptible at the time of recording, as it was a quiet area, even considering that was located in a crowded city. Likewise, for this type of scenario, there was a greater concentration of responses around the median for human and animal sounds, being higher than for the rest of the sources. This is in accordance with a study that evaluated the perception of noise sources in urban open spaces by Yang and Kang, who concluded that the overall evaluation of the soundscape is greatly affected by the sound source type [6]. 

In this regard, when assessing H1, all the sound sources were perceived as more pleasant WOH, probably because helicopter noise masked them partially. Significance in the differences occurred between most pairs of variables and, in particular, for all the sound sources compared at L2. 

When comparing how the helicopter noise was perceived at paired locations (H2), at L1, the helicopter noise was considered more pleasant than at L3, even if both locations were at similar distances. Two aspects could have influenced this difference. The first one is the type of noise, since, at L1, the helicopter was at the ground idle phase (less noisy). The second one is related to the directivity pattern of the helicopter noise: according to the study of Fastl et al. [31], the loudness and sharpness on the sides are greater than in front and behind the tail rotor (which explains why the ratings for the helicopter noise are worse at L2). In addition, the helicopter noise is perceived to be sharper in front than at the rear of the helicopter. In the present study, sharpness was the psychoacoustic parameter better correlated to the subjective evaluations of the soundscape. This, together with a lower noise level, probably explains why people preferred location L1. However, at L3, for more than half of the playback duration, the noise from the helicopter was no longer noticeable as the helicopter took off and moved away from the heliport facility, and yet the noise from the helicopter was considered less pleasant in this scenario (H4); moreover, not only the noise of the helicopter, but, in general, the soundscape was considered less appropriate at L3 than at L1 (H6). Therefore, the type of noise, for the same distance, seems to be more decisive in the soundscape evaluation than the duration of the helicopter noise.

When evaluating the soundscape using SDS and the violin plots, the scenarios WOH were better rated than the others for the adjectives with positive connotations (pleasant, uneventful, and calm). The opposite happened for adjectives with negative connotations (unpleasant, eventful, chaotic), although no clear trend could be identified for monotonous and exciting. When evaluating the differences in the semantical differential scale ratings (H3) using the Wilcoxon signed-rank test, for the SDS-Monotonous and SDS-Eventful, there were no statistically significant differences between the scenarios WH and WOH. The study of Kogan et al. [44] applied the model of Axelsson et al. to classify different urban open spaces, 21 in Latin America and 8 in Europe, leading to the conclusion that the term “monotonous” was not used to define, on average, any of the areas. This happened probably because it was an adjective that did not fit well with the characteristics of the soundscapes evaluated, as may have been the case in our study.

**Figure 8:**
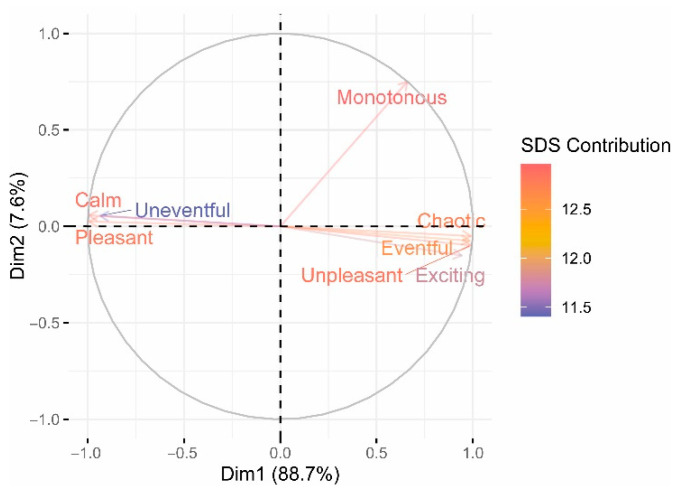
Component scores of the 8 semantic differential scales under study. Red to blue scales were used to represent the contribution of each variable to the model.

**Figure 9:**
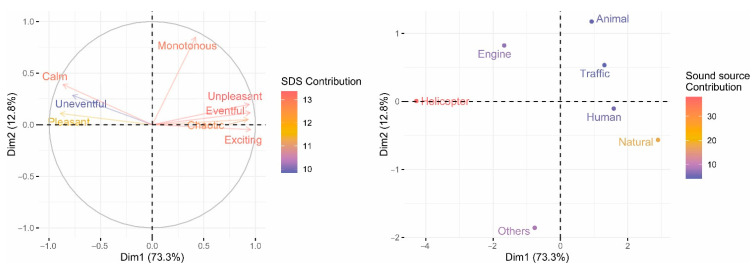
Component scores of the 8 semantic differential scales (SDS) (**left**) using the noise sources as loading scores (**right**). Blue to red scales were used to represent the contribution of each variable.

To further investigate the role played by the different SDS in defining the characteristics of the soundscape, two principal component analyses have been conducted. For the first of them, only the associations between the SDS have been used as input data (Figure 8). For the second, the pleasantness of the noise sources has been set as the model load (Figure 9). 

The loading plot (left) represents how strongly each variable influences a principal component. The (0,0) point represents the average value for each variable. The arrow shows the loading information and indicates how well a variable is represented by the graph. Non-correlation leads to two vectors out of phase by 90°.

For the first model, the first and the second principal components explain 96.3% of the variance (Figure 8). The scales mostly contributing to the first PC are Calm and Pleasant (20%), followed by Uneventful and Unpleasant (12% and 10%, respectively), whereas the highest contributor to the second component is Monotonous (74%). This factor behaves differently to the others, regarding the size of the participation and the direction of the vectors. It is worth highlighting than Monotonous is a unique variable that presents a low or non-significant correlation with the other SDS (see Figure 8 and Appendix B); this may be one of the reasons that it is isolated in the figure, as it is able to explain information not contained in other variables. Calm, Uneventful, and Pleasant are positively correlated with one another, as also occurs with Unpleasant, Eventful, Chaotic, and Exciting. However, both groups of scales are negatively correlated with each other, since the vectors that represent them diverge. This involves a different behavioral trend of the factor Exciting too, which was manifested in the two-dimensional model for the soundscape of Axelsson et al. [5], in which Exciting was viewed as a combination of Pleasantness and Eventfulness (and, in the present study, was closer to Eventfulness, and opposite to Pleasantness). Furthermore, the axes corresponding to the Pleasantness and the Eventfulness dimensions are not perpendicular—as in the mentioned Axelsson et al. model. In our case, there is only a main principal direction that gathers two groups of adjectives at the extremes with divergent loadings, at angles of 180° approximately. We will outline several factors that may be leading to these differences. Semantic differences between the language of the original study and Ecuadorian [5] may have given rise to the use of terms that are not usually associated with the acoustic environment, even though the translation was carried out by Ecuadorians. Imperfectly paired matched antonyms may also have influenced the differences between both studies. In our study, all the scenarios were experienced by 26 subjects, and in the study of Axelsson, each scenario was assessed by 10 subjects (although there were 50 excerpts, not all participants listened to all the excerpts), so we can disregard the number of participants as a possible cause of the different results. However, Axelsson et al. tried to explain their model through the large variability of acoustic scenarios (although scenarios with helicopter noise were not evaluated), and we focused only on two types (WH and WOH), so we could state that ours is a noise-source-specific model. Another possible reason is related to the distracting effect of the visual stimuli on the results, as our study was based on audio-visual scenarios and the study of Axelsson et al. on audio-only scenarios. Although we opt for these last two reasons as the ones that may be motivating the differences, more research is needed to clarify what is really happening.

For the second model, principal components PC1 and PC2 contribute to 86% of the variance (Figure 9). The contribution to PC1 of Calm, Exciting, Eventful, and Chaotic is very similar and close to 15%. Uneventful, Pleasant, and Calm present a contribution of 10%, 13%, and 14%, respectively. The scales that contribute the most to PC2 are Monotonous and Calm, with 70% and 15%, respectively. The strong influence of the helicopter noise on the overall results (Figure 9, right) may be influencing the anomalous gathering of two groups (similar to that of the first model), one with positive and the other with negative connotations.

Loudness represents the subjective perception of auditory characteristics linked to intensity sensation [45], while sharpness is an evaluation of the high-frequency content of the sound [29,30], associated with sensory unpleasantness [46]. In the present study, the psychoacoustic parameters that show the highest correlation with the pleasantness of the acoustic environment are Loudness (r = −0.25) and Sharpness (r = −0.49) (H7). Sharpness also shows moderate correlations with most subjective parameters. These results coincide with the statements of Fastl et al. [45], who argue that loudness and sharpness are inversely related to sensory pleasantness. In this regard, Boucher et al. [47] conducted a multilevel regression analysis on helicopter noise perception, identifying sharpness as an important factor for predicting annoyance. It is also in line with the results of the laboratory test conducted by Krishnamurthy et al. [48], in which 105 helicopter sounds served as the acoustic stimuli for the evaluation of different psychoacoustic parameters, leading to the conclusion that sharpness is the most influential psychoacoustic parameter when describing annoyance caused by helicopter noise. It is worth highlighting that, for both research works, loudness was a controlling factor with sparse variability (slightly below a noticeable difference), so they were not able to evaluate its influence on the perception of the helicopter noise.

Roughness and fluctuation strength reflect the temporal changes of sounds; they are related to slow (below 20 Hz) and fast sound variations, respectively [49]. This is probably the reason that the highest correlation coefficient of both variables occurred with the SDS-Eventfulness.

## 5. Conclusions

The present study evaluates the relationship between appraisals of the soundscape and the presence or absence of helicopter noise in the vicinity of a heliport, using qualitative scales of soundscape appraisal. In the laboratory test, 360-degree videos with spatial audio recorded in the Parque del Bicentenario of Quito, Ecuador, were shown to the participants using the Oculus headset. Seven hypotheses were formulated to assess statistically significant differences between the scenarios WH and WHO and between locations. The main findings can be summarized according to the formulated hypotheses.

**C1.**  *All the sound sources are considered more pleasant in the scenarios WHO than in those WH.*

**C2.**  *No clear conclusions can be inferred from the perceived differences of the sound sources between locations (since the differences between them are not statistically significant), except for the aircraft category; when using two types of helicopter noise at similar distances, the helicopter noise of the location with the ground idle operational phase is considered more pleasant than the other. This happens even if the duration of the flight idle noise lasts for only half the audio-visual scenario.*

**C3.**  *For the semantic differential scales considered positive, the scenarios WOH are better rated than the others for the adjectives with positive connotations (pleasant, uneventful, and calm). The opposite happens for adjectives with negative connotations (unpleasant, eventful, chaotic), although no clear trend can be identified for monotonous and exciting. The evaluation of the different SDS using principal component analyses revealed appreciable differences in the axes’ distribution from the original two-dimensional model of Axelsson et al. Further research is needed to determine the possible reasons for these differences.*

**C4.**  *No clear trend could be extracted for all the SDS regarding the differences between locations. However, for the locations with different types of helicopter noise, the soundscape of the location with the ground idle operational phase was considered more pleasant and calm than the others.*

**C5.**  *All the scenarios WOH were considered more appropriate and with a better landscape quality. The intention to visit the park was lower if the expected acoustic scenarios were WH.*

**C6.**  *The soundscape was considered less appropriate at the locations with the flight idle noise. A similar case was noted with the landscape quality, although the differences were not always statistically significant. At locations with the same type of helicopter noise, higher noise levels lead people to consider the soundscape less appropriate.*

**C7.**  *The strongest associations between objective and subjective parameters emerged between LZ_eq_, LA_eq_, LC_eq_, and the SDS Pleasant and Calm. However, the role of sharpness, in accordance with other studies, has been shown to have great importance in the soundscape perception, being its prediction a key aspect of the heliport facilities’ design.*

This study allows the first steps to be taken in the development of design criteria for heliports in urban parks, which establish, for example, minimum separation distances between stay areas of urban parks and helipads.

## Figures and Tables

**Table 1:** Age and gender of the participants, split into the percentage of people who gave negative or neutral and positive ratings to the appropriateness of the soundscape for the three locations with (WH) and without the helicopter noise (WOH). The number of responses is given in parentheses. As there were 3 locations, responses are the number of participants multiplied by three for both the scenarios with and without helicopter noise. In addition, 100% refers to the total number of responses.

		Ratings Given to the Appropriateness of the Soundscape			
	Number of Participants	With (WH)		Without (WOH)	
		Negative or Neutral	Positive	Negative or Neutral	Positive
Gender	Woman//13	25.64% (20)	24.36% (19)	6.41% (5)	43.59% (34)
	Man//13	41.03% (32)	8.97% (7)	5.12% (4)	44.87% (35)
Age	18–24//9	25.64% (20)	8.97% (7)	3.85% (3)	30.77% (24)
	25–34//7	17.95% (14)	8.97% (7)	3.84% (3)	23.08% (18)
	35–44//2	3.85% (3)	3.85% (3)	0.00% (0)	7.69% (6)
	45–54//2	3.84% (3)	3.85% (3)	1.28% (1)	6.41% (5)
	55–66//6	15.39% (12)	7.69% (6)	2.56% (2)	20.51% (16)

**Table 2:** Wilcoxon signed-rank test results (*p*-value and comparison of ratings between paired variables) of the appraisals of the sound sources (sounds of road traffic, aircraft, engines, humans, animals, naturals, and others) for the scenarios with (WH) and without helicopter noise (WOH). Results are shown for the three locations under study (L1, L2, and L3). For the analysis of the participants’ appraisals, “a” is the first, and “b” is the second variable of the pair.

Sound Source Category	Location	a	b	Wilcoxon *p*-Value	Lower (a < b)	Equal (a = b)	Higher (a > b)	Effect Size
Traffic	L1	WOH	WH	0.014	4	9	13	0.483
	L2	WOH	WH	0.000	0	9	17	0.714
	L3	WOH	WH	0.131	6	11	9	0.296
Aircraft	L1	WOH	WH	0.049	6	4	16	0.386
	L2	WOH	WH	0.000	2	1	23	0.728
	L3	WOH	WH	0.000	3	2	21	0.783
Engine	L1	WOH	WH	0.031	5	7	14	0.423
	L2	WOH	WH	0.000	2	4	20	0.728
	L3	WOH	WH	0.008	3	11	12	0.519
Human	L1	WOH	WH	0.023	8	1	17	0.445
	L2	WOH	WH	0.011	3	10	13	0.500
	L3	WOH	WH	0.022	5	8	13	0.450
Animal	L1	WOH	WH	0.049	5	10	11	0.386
	L2	WOH	WH	0.000	1	8	17	0.721
	L3	WOH	WH	0.036	5	5	16	0.410
Natural	L1	WOH	WH	0.187	9	4	13	0.259
	L2	WOH	WH	0.000	0	8	18	0.735
	L3	WOH	WH	0.938	7	10	9	0.015
Other	L1	WOH	WH	0.097	8	2	16	0.326
	L2	WOH	WH	0.000	2	7	17	0.692
	L3	WOH	WH	0.003	3	7	16	0.589

**Table 3:** Wilcoxon signed-rank test results (*p*-value and comparison of ratings between paired variables) for the scenarios with (WH) and without helicopter noise (WOH) on the appraisals given to each SDS (pleasant, uneventful, calm, monotonous, unpleasant, uneventful, chaotic, and exciting). Results are shown for the three locations under study (L1, L2, and L3). For the analysis of the participants’ appraisals, “a” is the first, and “b” is the second variable of the pair.

SDS	Location	a	b	Wilcoxon *p*-Value	Lower (a < b)	Equal (a = b)	Higher (a > b)	Effect Size
Pleasant	L1	WOH	WH	0.000	1	5	20	0.713
	L2	WOH	WH	0.000	0	3	23	0.834
	L3	WOH	WH	0.000	1	2	23	0.830
Uneventful	L1	WOH	WH	0.069	6	7	13	0.357
	L2	WOH	WH	0.001	3	2	21	0.646
	L3	WOH	WH	0.027	7	3	16	0.433
Calm	L1	WOH	WH	0.000	2	8	16	0.706
	L2	WOH	WH	0.000	0	1	25	0.885
	L3	WOH	WH	0.000	2	5	19	0.750
Monotonous	L1	WOH	WH	0.039	15	2	9	0.393
	L2	WOH	WH	0.218	12	7	7	0.242
	L3	WOH	WH	0.348	10	8	8	0.184
Unpleasant	L1	WOH	WH	0.000	20	3	3	0.746
	L2	WOH	WH	0.000	24	1	1	0.860
	L3	WOH	WH	0.000	18	6	2	0.719
Eventful	L1	WOH	WH	0.000	22	3	1	0.759
	L2	WOH	WH	0.000	25	0	1	0.867
	L3	WOH	WH	0.001	19	3	4	0.646
Chaotic	L1	WOH	WH	0.001	15	9	2	0.652
	L2	WOH	WH	0.000	23	3	0	0.842
	L3	WOH	WH	0.003	18	2	6	0.591
Exciting	L1	WOH	WH	0.007	16	6	4	0.532
	L2	WOH	WH	0.000	20	5	1	0.752
	L3	WOH	WH	0.023	16	6	4	0.446

**Table 4:** Wilcoxon signed-rank test results (*p*-value and comparison of ratings between paired variables) for the scenarios with (WH) and without helicopter noise (WOH) on the appraisals given to the soundscape appropriateness, the visual quality, and the frequency of visits. Results are shown for the locations L1 (red), L2 (green), and L3 (blue). Significant (continuous line) and non-significant differences (dashed line) between paired variables are also represented.

General Appraisal	Location	a	b	Wilcoxon *p*-Value	Lower (a < b)	Equal (a = b)	Higher (a > b)	Effect Size
Soundscape appropriateness	L1	WOH	WH	0.014	5	4	17	0.599
	L2	WOH	WH	0.000	1	2	23	0.806
	L3	WOH	WH	0.031	1	4	21	0.838
Landscape quality	L1	WOH	WH	0.049	5	8	13	0.759
	L2	WOH	WH	0.000	2	5	19	0.436
	L3	WOH	WH	0.000	1	8	17	0.722
Frequency of visits	L1	WOH	WH	0.031	2	3	21	0.616
	L2	WOH	WH	0.000	4	12	10	0.154
	L3	WOH	WH	0.008	0	4	22	0.809

**Table 5:** Spearman’s correlation coefficients between the subjective ratings on the acoustic environment (sound sources, SDS, soundscape appropriateness, landscape quality, and frequency of visits) and the objective acoustic parameters (Leq, LAeq, LCeq, Loudness, Sharpness, Roughness, and Fluctuation Strength). The *p*-values are in parentheses. Large association between variables according to Cohen’s criterion, is marked in bold. * The correlation is significant at the 0.05 level. ** The correlation is significant at the 0.01 level.

		Loudness	Sharpness	Fluctuation Strength	Roughness	LZeq	LCeq	LAeq
Sound sources	Traffic	−0.03 (0.75)	−0.19 * (0.02)	0.15 (0.06)	0.12 (0.12)	−0.31 ** (0.00)	−0.34 ** (0.00)	−0.34 ** (0.00)
	Aircraft	−0.22 ** (0.01)	−0.32 ** (0.00)	0.01 (0.92)	0.02 (0.78)	−0.58 ** (0.00)	−0.59 ** (0.00)	−0.59 ** (0.00)
	Engine	−0.11 (0.18)	−0.23 ** (0.00)	0.07 (0.37)	0.05 (0.55)	−0.46 ** (0.00)	−0.48 ** (0.00)	−0.48 ** (0.00)
	Human	−0.14 (0.07)	−0.15 (0.06)	0.02 (0.78)	0.01 (0.91)	−0.28 ** (0.00)	−0.27 ** (0.00)	−0.27 ** (0.00)
	Animal	−0.11 (0.19)	−0.22 ** (0.01)	0.12 (0.14)	0.16 * (0.05)	−0.41 ** (0.00)	−0.44 ** (0.00)	−0.44 ** (0.00)
	Natural	0.06 (0.48)	−0.20 * (0.01)	0.21 * (0.01)	0.13 (0.10)	−0.25 ** (0.00)	−0.31 ** (0.00)	−0.31 ** (0.00)
	Others	−0.13 (0.11)	−0.25 ** (0.00)	0.05 (0.57)	0.07 (0.37)	−0.39 ** (0.00)	−0.41 ** (0.00)	−0.41 ** (0.00)
SDS	Pleasant	−0.25 ** (0.00)	−0.49 ** (0.00)	0.12 (0.13)	0.15 (0.05)	−0.67 ** (0.00)	−0.71 ** (0.00)	−0.71 ** (0.00)
	Chaotic	0.12 (0.13)	0.41 ** (0.00)	−0.17 * (0.04)	−0.11 (0.19)	0.56 ** (0.00)	0.61 ** (0.00)	0.61 ** (0.00)
	Exciting	0.10 (0.22)	0.36 ** (0.00)	−0.13 (0.12)	−0.07 (0.38)	0.42 ** (0.00)	0.46 ** (0.00)	0.46 ** (0.00)
	Uneventful	−0.11 (0.18)	−0.25 ** (0.00)	0.09 (0.29)	0.04 (0.66)	−0.45 ** (0.00)	−0.47** (0.00)	−0.47 ** (0.00)
	Calm	−0.20 * (0.01)	−0.45 ** (0.00)	0.12 (0.15)	0.11 (0.19)	−0.70 ** (0.00)	−0.74 ** (0.00)	−0.74 ** (0.00)
	Unpleasant	0.17 * (0.03)	0.42 ** (0.00)	−0.13 (0.09)	−0.11 (0.19)	0.57 ** (0.00)	0.61 ** (0.00)	0.61 ** (0.00)
	Eventful	0.10 (0.20)	0.45 ** (0.00)	−0.25 ** (0.00)	−0.24 ** (0.00)	0.59 ** (0.00)	0.66 ** (0.00)	0.66 ** (0.00)
	Monotonous	0.06 (0.49)	0.13 (0.10)	−0.06 (0.43)	−0.09 (0.26)	0.13 (0.11)	0.15 (0.06)	0.15 (0.06)
General	Appropriateness	−0.15 (0.06)	−0.40 ** (0.00)	0.13 (0.10)	0.12 (0.15)	−0.60 ** (0.00)	−0.65 ** (0.00)	−0.65 ** (0.00)
	Visual	−0.12 (0.13)	−0.24 ** (0.00)	0.05 (0.56)	0.01 (0.88)	−0.40 ** (0.00)	−0.41 ** (0.00)	−0.41 ** (0.00)
	Frequency	−0.15 (0.06)	−0.39 ** (0.00)	0.12 (0.12)	0.07 (0.35)	−0.61 ** (0.00)	−0.65 ** (0.00)	−0.65 ** (0.00)

**Figure A1:**
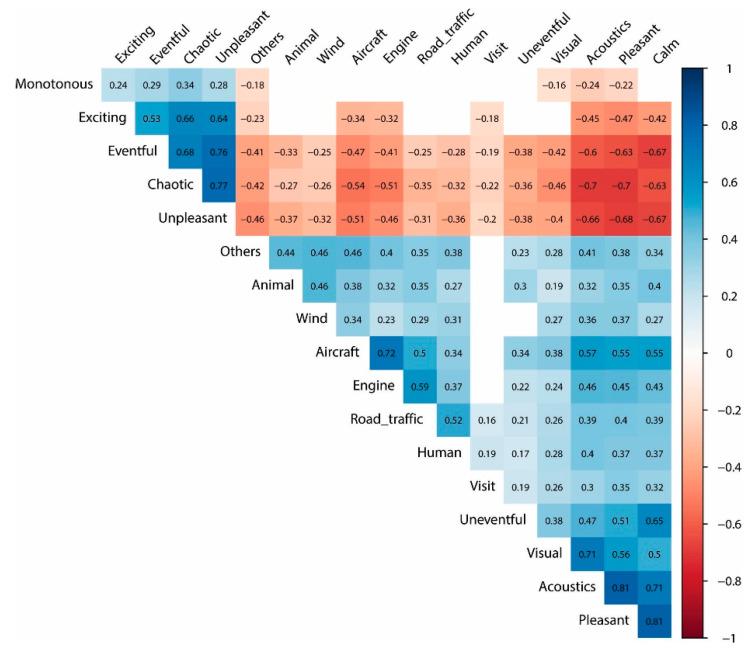
Spearman’s correlation coefficients between the subjective ordinal variables of the study.

## Data Availability

The data presented in this study are available on justified request to the corresponding author. The data are not publicly available due to privacy reasons.

## Appendix A

The questions conducted during the second part of the laboratory test are listed in Table A1.

**Table A1:** Questions asked to participants using a head-mounted display.

Questions	Measurement Scale
How pleasant or unpleasant the following sound sources are in this scenario? (¿Cómo de agradables o desagradables son los siguientes sonidos en este escenario?)	Very unpleasant (−100), neutral (0), Very unpleasant (100)
Traffic (e.g., light and heavy vehicles), Aircraft (e.g., overflying planes, helicopters…), Machinery engines (e.g., construction machinery, electric generators…), Human (e.g., steps, laughter, conversations…), Natural (e.g., wind, rain…), Animal (e.g., barks, birds singing, croaking fogs) and Other sounds.	
Tráfico (ligero, pesado…), Aviones (aviones pasando, helicópteros), Motores de maquinaria (maquinaria de construcción, generadores eléctricos…), Humanos (pasos, risas, conversaciones…), Natural (viento, lluvia…), Animal (ladridos, pájaros cantando, ranas croando…) y Otros	
2. How do you judge theacoustic environment of this scenario in relation to the following adjectives? (¿Cómo juzgas el ambiente acústico de este escenario en relación a los siguientes adjetivos?)	Nothing at all (0), Completely (100)
Scales: Pleasant, Chaotic, Exciting, Eventful, Unpleasant, Calm, Uneventful, Monotonous	
Escalas: Agradable, Caótico, Vibrante, Con pocos eventos, Irritante, Tranquilo, Con muchos eventos, Monónoto	
3. How appropriate is this acoustic environment to this place? (¿Cómo de apropriado es este ambiente acústico para este lugar?)	Completely inappropriate (−100), Neutral (0), Completely appropriate (100)
4. How would you describe the quality of the present landscape? (¿Cómo describirías la calidad de este paisaje?)	Very bad (−100), Not bad-not good (0), Very good (100)
5. If the conditions were the ones of this scenario, how frequently would you visit the park? (Si las condiciones fueran como las de este escenario, ¿con qué frecuencia visitaría el parque?)	Never (0), Very frequently (100)

## Appendix B

Table A2 shows the participants’ responses regarding the pleasantness of the sound sources split by the number of non-positive (negative or neutral) and positive ratings of the soundscape quality and by the number of non-positive (negative or neutral) and positive ratings of the landscape quality, with (WH) and without the helicopter noise (WOH). The number of responses is given in parentheses. As there are three locations, the number in parentheses is the number of responses given for the three locations (L1, L3, and L3), considering the scenarios WH and WOH. Considering that the desirable appraisals of the soundscape of a park would be positive, the responses have been divided into two main groups that consider non-positive (negative and neutral) and positive appraisals.

**Table A2:** Participants’ responses to the pleasantness of the sound sources, split by the number of non-positive (negative or neutral) and positive ratings of the appropriateness of the soundscape (AP) and by the number of non-positive (negative or neutral) and positive ratings of the landscape quality (VQ), with and without the helicopter noise. As there are 3 locations, the total number of responses is the number of participants multiplied by three for both the scenarios with and without helicopter noise. The number of responses is given in parentheses. The highest percentages of participants are marked in bold. In addition, 100% refers to the total number of responses.

		With Helicopter Noise				Without Helicopter Noise			
Sound Source	Rating	AP Appraisal		VQ Appraisal		AP Appraisal		VQ Appraisal	
		Negative or Neutral	Positive	Negative or Neutral	Positive	Negative or Neutral	Positive	Negative or Neutral	Positive
Road	Negative or neutral	**57.7%** **(45)**	24.36% (19)	**42.3%** **(33)**	39.75% (31)	10.25% (8)	**50%** **(39)**	8.97% (7)	**51.28%** **(40)**
	Positive	8.97% (7)	8.97% (7)	7.7% (6)	10.26% (8)	1.28% (1)	38.46% (30)	2.56% (2)	37.18% (29)
Aircraft	Negative or neutral	**64.1%** **(50)**	19.23% (15)	**47.44%** **(37)**	35.9% (28)	8.97% (7)	**53.84%** **(42)**	7.69% (6)	**55.13%** **(43)**
	Positive	2.56% (2)	14.1% (11)	2.56% (2)	14.1% (11)	2.56% (2)	34.62% (27)	3.85% (3)	33.33% (26)
Engines	Negative or neutral	**60.26%** **(47)**	23.07% (18)	**44.87%** **(35)**	38.46% (30)	7.69% (6)	**64.1%** **(50)**	7.69% (6)	**64.1%** **(50)**
	Positive	6.41% (5)	10.26% (8)	5.12% (4)	11.54% (9)	3.84% (3)	24.36% (19)	3.85% (3)	24.36% (19)
Humans	Negative or neutral	**60.25%** **(47)**	16.67% (13)	**41.03%** **(32)**	35.89% (28)	7.68% (6)	38.46% (30)	7.69% (6)	38.46% (30)
	Positive	6.41% (5)	16.67% (13)	8.98% (7)	14.1% (11)	3.85% (3)	**50%** **(39)**	3.85% (3)	**50%** **(39)**
Animals	Negative or neutral	**57.69%** **(45)**	26.93% (21)	**46.15%** **(36)**	38.46% (30)	3.84% (3)	32.06% (25)	3.85% (3)	32.05% (25)
	Positive	8.97% (7)	6.41% (5)	3.84% (3)	11.54% (9)	7.69% (6)	**56.41%** **(44)**	7.69% (6)	**56.41%** **(44)**
Wind	Negative or neutral	**53.85%** **(42)**	11.54% (9)	**34.62%** **(27)**	30.77% (24)	6.41% (5)	37.18% (29)	3.85% (3)	39.74% (31)
	Positive	12.82% (10)	21.79% (17)	15.39% (12)	19.23% (15)	5.13% (4)	**51.28%** **(40)**	7.69% (6)	**48.72%** **(38)**
Others	Negative or neutral	**57.69%** **(47)**	26.93% (13)	**43.6%** **(34)**	33.33% (26)	6.41% (5)	41.03% (32)	5.13% (4)	42.31% (33)
	Positive	8.97% (5)	6.41% (13)	6.41% (5)	16.67% (13)	5.13% (4)	**47.44%** **(37)**	6.41% (5)	**46.15%** **(36)**

Results show that when the helicopter is on, most participants give negative or neutral appraisals to all the sound sources, and also negative or neutral appraisals to both the soundscape (>53% for all the sound sources) and the landscape quality (>41%). When the helicopter sounds cannot be heard, also a higher number of participants give negative ratings to the “artificial” sound sources (traffic, aircraft, and engine noises), but positive ratings to the others (humans, animals, wind, and other noises) (≥50% for all the sound sources). For this last condition (WOH), although most people give negative or neutral ratings to the artificial sounds, the soundscape is considered pleasant by a high percentage of participants, which indicates that, for them, these “not wanted” sounds [50] do not influence the overall rating of the soundscape. Although the landscape features did not change for the scenarios with and without the helicopter noise, for the scenarios WOH, 88.46% gave positive appraisals of the landscape quality, and for scenarios WH, only 50% did so.

Table A3 shows the number of people who gave negative or neutral and positive ratings of the appropriateness of the soundscape and the landscape quality of the three locations WH and WOH. The number of responses is given in parentheses. As there are three locations, the number in parentheses is the number of responses given for the three locations (26 × 3), considering the scenarios with and without helicopter noise. Only neutral and non-neutral ratings have been considered as unipolar semantic scales were evaluated.

**Table A3:** Percentage of neutral and non-neutral ratings given to the semantic differential scales, split by the percentage of non-positive (negative or neutral) and positive ratings of the soundscape appropriateness (AE appraisal) and the landscape quality (VE appraisal) with and without the helicopter noise. As there are 3 locations, the total number of responses is the number of participants multiplied by three for both the scenarios with and without helicopter noise. The number of responses is given in parentheses. The highest percentages of participants are marked in bold. In addition, 100% refers to the total number of responses.

		With Helicopter Noise				Without Helicopter Noise			
SDS Scale	SDS Appraisal	SA Appraisal		VE Appraisal		SA Appraisal		VE Appraisal	
		Negative or Neutral	Positive	Negative or Neutral	Positive	Negative or Neutral	Positive	Negative or Neutral	Positive
Pleasant	Neutral	**42.31%** **(33)**	7.69% (6)	21.79% (17)	28.2% (22)	1.28% (1)	1.28% (1)	1.28% (1)	1.28% (1)
	Not neutral	16.67% (13)	33.33% (26)	7.69% (6)	**42.31%** **(3)**	1.28% (1)	**96.15%** **(75)**	2.56% (2)	**94.87%** **(4)**
Chaotic	Neutral	2.56% (2)	14.1% (11)	0% (0)	16.66% (13)	0% (0)	**69.23%** **(54)**	1.28% (1)	**67.95%** **(53)**
	Not neutral	**56.42%** **(44)**	26.92% (21)	29.48% (23)	**53.84%** **(42)**	2.56% (2)	28.2% (22)	2.56% (2)	28.21% (22)
Exciting	Neutral	5.13% (4)	11.54% (9)	1.28% (1)	15.38% (12)	2.56% (2)	**53.85%** **(42)**	3.85% (3)	**52.56%** **(41)**
	Not neutral	**53.84%** **(42)**	29.48% (23)	28.21% (22)	**55.13%** **(43)**	0% (0)	43.59% (34)	0% (0)	43.59% (34)
Uneventful	Neutral	**38.46%** **(30)**	17.94% (14)	21.79% (17)	**34.61%** **(27)**	1.28% (1)	11.53% (9)	1.28% (1)	11.54% (9)
	Not neutral	20.51% (16)	23.08% (18)	7.69% (6)	35.9% (28)	1.28% (1)	**85.9%** **(67)**	2.56% (2)	**84.62%** **(66)**
Calm	Neutral	**48.72%** **(38)**	17.95% (14)	26.92% (21)	**39.74%** **(31)**	2.56% (2)	2.56% (2)	2.56% (2)	2.56% (2)
	Not neutral	10.25% (8)	23.08% (18)	2.56% (2)	30.77% (24)	0% (0)	**94.87%** **(74)**	1.28% (1)	**93.59%** **(73)**
Unpleasant	Neutral	2.56% (2)	14.1% (11)	0% (0)	16.67% (13)	1.28% (1)	**70.52%** **(55)**	2.56% (2)	**69.23%** **(54)**
	Not neutral	**56.41%** **(44)**	26.93% (21)	29.48% (23)	**53.85%** **(42)**	1.28% (1)	26.93% (21)	1.28% (1)	26.92% (21)
Eventful	Neutral	1.28% (1)	2.56% (2)	0% (0)	3.85% (3)	0% (0)	46.16% (36)	1.28% (1)	44.87% (35)
	Not neutral	**57.69%** **(45)**	38.46% (30)	29.49% (23)	**66.67%** **(52)**	2.56% (2)	**51.28%** **(40)**	2.56% (2)	**51.28%** **(40)**
Monotonous	Neutral	8.97% (7)	5.13% (4)	3.85% (3)	10.25% (8)	0% (0)	17.94% (14)	1.28% (1)	16.67% (13)
	Not neutral	**50%** **(39)**	35.89% (28)	25.64% (20)	**60.26%** **(47)**	2.56% (2)	**79.48%** **(62)**	2.56% (2)	**79.49%** **(62)**

For the scenarios in which the helicopter noise could be perceived, the highest number of participants who gave neutral ratings of the pleasantness (42.31%) gave also negative ratings of the soundscape quality. However, when considering the landscape appraisals, a high number of participants (42.31%) who gave positive ratings to the pleasantness of the soundscape gave also positive ratings to the quality of the landscape. For the SDS scales “chaotic”, “exciting”, “unpleasant”, “eventful”, and “monotonous”, most participants gave non-neutral ratings to the adjective and negative to the soundscape quality; these participants also gave positive ratings to the landscape quality.

For the scenario WOH, the number of participants who gave positive ratings was larger (96.15%).

Figure A1 shows the correlations between the ordinal variables used in the study, according to a color code. Red and blue squares mean negative and positive associations, respectively, between the variables. White squares involve a coefficient of significance higher than 0.05 and, consequently, no statistically significant association between variables. The command “hclust” was used for the hierarchical clustering of paired variables according to the type (positive or negative) and value of the association. The data regarding all the scenarios were included in the correlation matrix. A positive association was found between the paired combinations of the SDS-Pleasant and the different sound sources. A similar case was noted for SDS-Calm and SDS-Uneventful. Negative correlations could be found between paired combinations of the sound sources and the SDS with negative connotations (SDS-Unpleasant, SDS-Chaotic, and SDS-Eventful). The adjectives monotonous and exciting did not show significant correlations with any of the sound sources considered in the study.
